# Aligning agri-environmental-climate public good supply and desire in a sustainable Dutch agricultural sector

**DOI:** 10.1007/s13280-024-01983-z

**Published:** 2024-02-16

**Authors:** Atoesa S. Farokhi, Kina S. Harmanny, Catharina J. E. Schulp

**Affiliations:** Institute for Environmental Studies, Environmental Geography Group, Vrije Universiteit Amsterdam, De Boelelaan 1111, 1081HV Amsterdam, The Netherlands

**Keywords:** Ecosystem services, Explorative scenarios, Public goods, Stakeholder visions, Sustainable agriculture

## Abstract

European agricultural policies increasingly incorporate mechanisms for delivery of public goods. Sustainable public good delivery requires alignment between societal demand and landscape supply. However, the variation of demands or desires regarding future public good delivery among society is hardly known. We inventoried the desires for public goods across Dutch society, and projections of agricultural supply. A multi-method approach was used to find agreement levels between desired and expected change in good delivery. Most stakeholders expressed a desire for biodiversity and climate regulation from agriculture, whilst desire for natural heritage and recreation was less common. The utility of a public good to a stakeholder appears to influence its desire. Scenarios for agriculture focus on extensification, sustainable intensification, regionalization, or meadow bird conservation. Regionalized scenarios showed the highest agreement between public good supply and desire. Maximizing alignment between desire and supply thus requires a challenging transition towards region-specific agricultural sustainability strategies.

**Supplementary Information:**

The online version contains supplementary material available at 10.1007/s13280-024-01983-z.

## Introduction

Agriculture intrinsically modifies and impacts natural landscapes. Since the 1950s, the expansion and intensification of agriculture however has accelerated, resulting in increased rates of deforestation, pollution, and climate change (Springmann et al. 2018; Rust et al. 2021). Reconciling agricultural productivity with landscape aesthetics and biodiversity is increasingly difficult due to socio-agricultural trade-offs (Verkerk et al. 2018; García-Martín et al. 2021). As a result, the cultural value of agricultural areas is jeopardized and ecosystem integrity is at risk (García-Martín et al. 2021).

Increasing calls for ecosystem restoration aim to halt the global loss of ecosystem integrity (Quintero-Uribe et al. 2022). For example, the European Union (EU) legally committed towards restoring habitats, species and ecosystems on 20% of their land and sea area by 2030 (European Commission 2022a). Such restoration commitments can be conceptualized using land management assessment frameworks describing the society-nature interface (Quintero-Uribe et al. 2022). Specific to the agricultural realm, non-commodity supplies of agriculture that benefit society are often framed as agri-environmental-climate public goods (AECPGs) (Westhoek et al. 2013; Verkerk et al. 2018). AECPGs are non-exclusive and non-rival, although market failures arise when supply and demand of AECPGs do not align (Dwyer et al. 2015). Examples of AECPGs include biodiversity, aesthetic landscape quality and water quality.

Over the past years, several EU-level policy instruments aiming to increase the delivery of AECPGs have been implemented. Such instruments (e.g., financial compensation for land managers) are however targeted primarily towards biodiversity and water quality (Reed et al. 2022), and are considered unsatisfactory in terms of effectiveness (Tyllianakis and Martin-Ortega 2021) and impact (Westhoek et al. 2013). Effective and sustainable delivery of AECPGs means that the demands of current and future generations are fulfilled. Yet, the demand for specific AECPGs across society remains underexplored. AECPG demand is known to be region specific (Westhoek et al. 2013), and several studies inventoried the direct use (Wolff et al. 2015) of ecosystem services (García-Nieto et al. 2015; Zoderer et al. 2019). However, knowledge and understanding of how direct use of and desires regarding AECPGs varies across society lags behind. While the current expressed demand for AECPGs can be quantified, the actual future demand cannot be quantified. Therefore, we refer to this as *(expressed) desire* for AECPGs.

Ensuring long-term AECPG supply throughout Europe requires a transition of EU’s rural landscapes, but agreeing on the best transition pathway appears challenging (Helfenstein et al. 2022). Explorative scenario studies have provided *plausible* pathways for European rural landscapes (Verkerk et al. 2018) describing how they *might* evolve. Such studies remain ambiguous on what *should* happen. This is complicated by the different, often contradictory priorities of producers, consumers, governments and other stakeholders. Additionally, political weight of stakeholders’ voices is unequal due to deep rooted inequality in gender, age, and other power asymmetries (Bock 2015). Normative visions instead provide insight into desired futures of a broad range of stakeholders (Helfenstein et al. 2022). Integrating normative visions and explorative scenarios for EU’s rural landscapes might therefore be a solution to support credible and legitimate policies that foster long-term AECPG supply (Verkerk et al. 2018).

The agricultural sector of the Netherlands is facing major challenges related to biodiversity loss, pollution, land degradation, and climate change (see “Case study area” section) and it is widely agreed that a transformation of the sector is required (Gonzalez-Martinez et al. 2021). This transformation should consider long-term AECPG delivery to society, which might serve as a tool in transformation, by providing an alternative source of income (Westhoek et al. 2013). Incentivizing farmers to change their practices is a key challenge, that relies on developing and operationalizing viable business models (Helfenstein et al. 2022; Staghouwer 2022) that meet and monetize the demands for AECPGs across society.

Given the lack of insight in the variation of AECPG demand across society and the role of AECPGs in the transformation of the Dutch rural landscape, this paper aims to identify long-term targets for AECPG delivery in the Netherlands. We inventory a broad range of visions on desired future AECPG delivery among society, and inventoried how recent scenarios for the Dutch rural landscape could support AECPG delivery. To explore which scenario aligns best with societal visions, we calculate agreement between expressed AECPG desire and supply. Finally, the credibility of and pathway to the optimal scenario is discussed.

## Background

### Agri-environmental-climate public goods in society

AECPGs can be considered a means of interaction between agroecosystems and the societal environment. Risks to AECPGs delivery are anthropocentric, caused by drivers affecting the functioning of agroecosystems or by poor agroecosystem management (Schröter et al. 2019). Society responds to AECPG risks in different ways. Risks can be *mitigated*, by softening the drivers of agroecosystem degradation, like system overuse through intensification (van Lieshout et al. 2013), climate change or pollution (Kuiper et al. 2021). Society can also *adapt* to changes in AECPG delivery through e.g. diet alteration, AECPG substitution or recycling (Foley et al. 2011). Finally, *transformation responses* are strategies focused on agroecosystem management (Schröter et al. 2019), such as changes in farm style through national policies.

### Reflexivity

To be transparent about potential biases in data-collection (Berger 2015), we reflect on our positionality. The first author is a young, bicultural Dutch cis-woman, educated in a western university from post-positivist ontology. In this study she deemed the incorporation of pragmatic and feminist epistemologies applicable. Reality was hence approximated by examining social and practical experiences (definitions from Creswell 2013). E.g., by speaking to a women’s farm organization and an agroecological farm organization to explore experiences of marginalized stakeholders (selective sampling). Her mother language is Dutch, which allowed including a broad range of (non)academic sources. Being a Dutch citizen, she is part of the stakeholder group citizens, and aware that this creates a nested opinion on other stakeholders. However, the methodology minimizes this bias by using written vision statements. The coauthors of this paper are Dutch cis women with similar educational profiles. We aimed to take a gender lens into account where relevant and possible in this research.

### Case study area

The Dutch agricultural sector uses more than half of the land area in the Netherlands. The sector is dominated by intensive dairy (46 000, 29% of farms) and other intensive livestock (4000, 8%) farms, that used 65% of the farmland for grassland and fodder crops in 2020 (CBS 2023). 29% of the farmland is used for arable land, and 6% for horticulture. Between 2000 and 2020, the number of farmers decreased from 116 000 to 52 700, but the farmland area stayed more or less the same (CBS 2023). Only 3% of farms is mixed (CBS 2023). Agriculture contributes 1.4% of the country’s GNP, but the agri-food sector as a whole generates 6.4% of the countries’ GNP and is highly export-oriented (CBS 2023). The pig rearing, egg, and horticulture sectors are profitable, generating about twice or more the result than the average of €75 800 per farm. Grazing livestock (70%) and arable farming (82%) are less profitable.

Different government levels influence the Dutch agricultural sector. The EU directs national policy through, among others and most importantly, the Common Agricultural Policy (CAP). Nationally, policies and subsidy schemes are formulated by the Ministry of Agriculture, Nature and Food quality. Provinces implement agri-environmental measures and regional water boards govern water quality. Dutch (environmental) planning is known for its corporatist *Polder* model, valuing governance through cooperation and consensus building (Schreuder 2001). Including many stakeholders can enhance inclusivity, but balancing between their interests can favor compromises that hinder transformation.

Decades of deliberate intensification and scale enlargement have resulted in problematically high nitrogen deposition levels, endangered meadow bird populations, and low water quality. The dairy sector produces 85% of ammonia emission and 11% of GHG emissions and 60% of farms produce more manure than can be used on their own farm. Arable farms use high amounts of inorganic fertilizer (Schreefel et al. 2022). Soil P and N concentrations are very high (Panagos et al. 2022). Another threat to Dutch agriculture is the ongoing peat oxidation due the continuous draining that shaped 8% of the land area, resulting in 2.4–4.2% of Dutch GHG emissions (Poppe et al. 2021).

To comply with EU legislation on nature protection and water quality, a stringent nitrogen reduction plan was presented in June 2022 (Staghouwer 2022). In response, large-scale farmer protests arose, resulting in a heavily polarized debate about the future of Dutch agriculture (NOS Nieuws 2022). The Dutch government acknowledged the importance of AECPG delivery and admitted their responsibility in developing business models for AECPG delivery, but also expect engagement of value chains and citizens, demonstrated by involving a range of stakeholders in the development of the national agricultural transition policy (Staghouwer 2022). Yet, only the conventional and powerful (as described by Williams et al. 2023) stakeholders of the agri-food sector were included. Three conventional agriculture organizations are represented in the lobby register of the House of Representatives, and no alternative voices ((House of Representatives (Tweede Kamer) 2023). Between October 1st and December 31st 2022, the Dutch minister of Agriculture supported the development of an agreement about the national agricultural transition policy with 55 appointments with representatives of the sector, including one representative of pioneering farms ((Rijksoverheid 2022)). Additionally, apprehension of the preferences and opportunities for AECPG supply by farmers is missing. However, as most farms currently provide limited AECPGs (Schröter et al. 2019), this is in line with the status quo.

Socially, Dutch rural development policy has been seemingly inclusive and gender-neutral, but mostly because gender has been trivialized and structurally ignored in the Dutch agricultural debate (Bock 2015). Farm inheritance and creation are less common for women than men, largely because women face challenges in gaining recognition as a farmer (Ball 2020). The need for women organizations (Ball 2020) and their call for increased awareness of women’s societal role and position (LTO Vrouw en Bedrijf 2022) shows continued inequality. This also becomes apparent in the European critique on the proposed CAP National Strategic Plan, that does not address gender (European Commission 2022b). Drastic demographic change is expected in the Dutch agricultural sector over the coming decades: ageing (Debonne et al. 2022), feminization, and shrinkage (Staghouwer 2022). It is unknown how these demographic changes will affect AECPG delivery.

## Materials and methods

### Overview

**Fig. 1:**
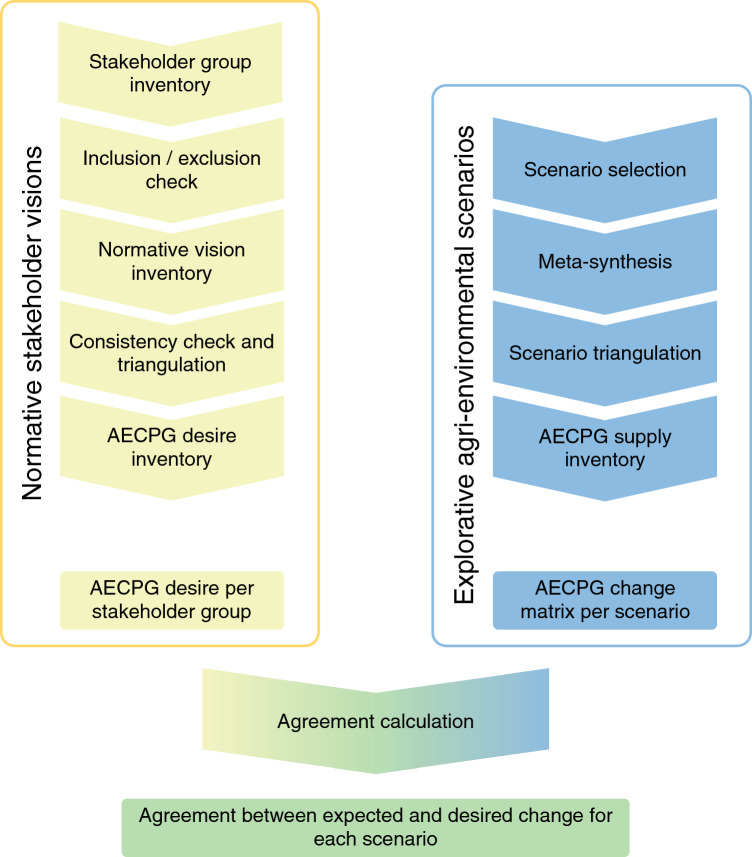
Methodological flowchart. On the left the creation of the expected change matrix using explorative scenarios is summarized. The right section shows the process of creating the desired change matrix using normative visions as input data. This method was adapted from Helfenstein et al. (2022)

Using a multi methods approach (Fig. 1), we explored and quantified agreement between desired and expected change in AECPG delivery. AECPGs included are biodiversity, aesthetic landscape quality, natural heritage, water quality, air quality, soil quality, recreation, quality of products and climate regulation (Table 1), based on their applicability in the Netherlands.

**Table 1:** Scoring table for AECPG desire. Each AECPG has different scoring criteria because of their broad range in functions and scales

AECPG	Synonyms	Synonyms (translation)	Assignment score (0)	Assignment score (1)
Biodiversity	Soortenrijkdom, agrobiodiversiteit	Species (richness), agrobiodiversity	There are no measures towards biodiversity enhancement mentioned or implied. No urgency is expressed	Enhancement or active conservation of species, ecosystem or genetic richness is expressed or clearly implied, in general or in case of specific species (e.g. meadow birds)
Aesthetic landscape quality	Landschaps-elementen, heggen	Landscape elements, hedgerows, scenic (not nature/natural)	Landscape is just mentioned as a given or nice to have	Specific management or increase in landscape elements is mentioned or clearly implied
Heritage	Natuurlijk erfgoed, grutto’s, weidevogels	Natural heritage, mentioning of a typical agricultural landscape/land use	Natural heritage just mentioned as a given or nice to have, or the focus is on aesthetic quality without grounding in heritage, or on recreation	Action is required to maintain heritage is desired or implied
Water quality Air quality Soil quality	Water vervuiling, schoon water, luchtvervuiling, schone lucht, bodemvervuiling, erosie, organische stof, watervast-houdend vermogen	Water pollution, clean water, air pollution, clean air, soil pollution, erosion, organic matter, retention capacity	Stakeholders expressed desire to keep current management. Clean water/air/soil is mentioned as nice to have or as self-evident (not as a point of action)	Improvement of water/air/soil quality is specifically mentioned or implied Essential function of soil is emphasized
Quality of products	Kwaliteitsproducten, hoogwaardige producten, veilig en gezond voedsel	High quality products, safe and healthy food, added value	Stakeholder does not express the need to maintain or improve the quality of products under future challenges	Prioritization of product quality
Recreation	Vrije tijd, recreatie, wandelen, fietsen	Leisure, walking, biking (not health care)	No extra/explicit recreation opportunities are implied or mentioned	Visions specifically ask for recreational opportunities
Climate regulation	Klimaat, emissies	Climate, emissions (in case it concerns reducing GHG emissions or storing GHG) (No adaptation measures that do not relate to GHG levels such as plant drought resilience)	Climate change is narrated as a ‘have to’ comply, inevitable emission reduction according to set targets	Strong concern of climate change is expressed. Responsibility for increased emission reduction and carbon storage are mentioned or implied

AECPG desire was defined as the expressed relevance of the provision of an AECPG to the stakeholder group. Stakeholder groups and their visions were inventoried through a web review, interviews and survey data (“Normative stakeholder visions: Desired AECPG matrix” section). This resulted in a desired change matrix, showing expressed desire by stakeholders for each AECPG. To explore expected AECPG supply, existing scenarios for Dutch agriculture and rural landscapes were meta-synthesized and scored (“Exploratory scenarios: Expected AECPG matrix” section). The supply and desire matrices were combined to calculate agreement (“Comparing expected and desired AECPG delivery” section).

### Normative stakeholder visions: Desired AECPG matrix

#### Stakeholder inventory

We first identified broad stakeholder groups, based on recent literature (Schulp et al. 2022; Williams et al. 2023), agricultural policies on European and Dutch scale (e.g. Common Agricultural Policy (CAP)) and news items from agricultural news outlet.^1^ Next, we used expert judgement supported by information on e.g., market share, outreach, or number of members, to determine stakeholders’ political, economic or ideological power in the Dutch agricultural sector, which was used as inclusion criterion. Specific stakeholders in each group were identified through a web literature review, following the method as proposed by Stansfield et al. (2016). Google search was used to collect data. Stakeholder-group-specific search terms and inclusion criteria were used to find individual stakeholders (see Supplementary material). For example, to find relevant NGOs, the Dutch translation of the search term ‘ngo agriculture nature’ was used, for Dutch banks ‘banks in the Netherlands’ etc. For each search term, the first five pages of results were scanned. In case a search term did not yield usable data (e.g. ‘estate owners’), another search term for the same stakeholder group was attempted (‘estate owner association’). We traced websites of individual stakeholders, which were scanned on their homepage, ‘about us’ section, and vision/mission statements. If stakeholders met the inclusion criteria, for each included stakeholder normative visions were collected.

#### Visions of stakeholder groups (except citizens)

Next, we collected visions of each identified stakeholder (except ‘citizens’). Web pages of included stakeholders describing (i) visions, (ii) sustainability approaches or (iii) news items directing towards manifests, (annual) reports and policies were evaluated against inclusion criteria. Visions had to be written in Dutch or English, published in 2018 or later, relate to the national agricultural system or forestry, and imply or mention at least one AECPG. When explicit vision documents were available, they were used. In case of absence of vision documents, policies and statements on AECPGs were used instead. Multiple sources were only included for one stakeholder when distinctly different AECPGs were covered, e.g., if the stakeholder had separate specific documents/web pages describing their visions on biodiversity and climate.

After selection, the expressed AECPG desire in each vision was scored (Table 1). The scoring was done for individual stakeholders. To reveal relative prioritization of AECPGs, weights were given to AECPGs, similar to Helfenstein et al. (2022). If an AECPG was mentioned or implied but not central to the vision, it was weighed “1”. Central AECPGs obtained weight “2”. Unmentioned or unimplied AECPGs got weight “0”. A sensitivity analysis was performed (“Comparing expected and desired AECPG delivery” section) and three experts independently scored a random selection of 25 percent of the desired change matrix to check the scoring and weighing consistency.

#### Citizens’ visions

Citizens’ desire for AECPG delivery was quantified using national-level data from EU mass survey data (Eurobarometers) published between 2015 and 2022 (European Union 2022). The A–Z list of Eurobarometers was scanned for relevant titles, including the AECPGs considered, but also broader themes such as food, climate, and the CAP. Relevant surveys were screened for specific questions useful to quantify AECPG desire; these were included in our analysis when showing desire for, prioritization of, willingness-to-pay for, or concern about current delivery of an AECPG. At least two questions were selected per AECPG (Supplementary material).

Answers were differentiated by gender (men and women), age group (15–24, 25–34, 35–44, 45–54, 55–64, 65–74, 75 +), political spectrum (left-center-right) and subjective urbanization (rural–urban, as reported by the respondent). AECPG desire of citizen groups was scored by qualitative interpretation of the survey. Contrary to other stakeholders, three levels were distinguished (0–0.5–1) because the data revealed a noticeable difference between (i) desire for active conservation or minor increase of an AECPG and (ii) a clearly observable desire of delivery.

#### Consistency check and triangulation

Standard errors of the mean score within each stakeholder group was used as an indicator of homogeneity of stakeholder visions within each group. To check consistencies and understand potential divergence in qualitative data, data or methods from different viewpoints should be compared (triangulation; (Olsen 2004)). In the current debate and policy development about agriculture in the Netherlands, the perspective of women is understudied (Bock 2015) and the perspective of pioneering farms is marginalized (see “Case study area” section). To include these voices, agroecological farmers, and women in the largest Dutch farm organization, were interviewed (see Fig. 1, scenario triangulation). The women (from LTO Vrouw en Bedrijf) provided information about their vision on AECPG supply and demand, their work as a women’s interest group, the agricultural sector and its future. The agroecological society (Toekomstboeren) provided information on their vision on future agriculture and public goods. The transcripts are available on request. The interviews were used for triangulation of the results and the discussion.

### Exploratory scenarios: Expected AECPG matrix

To collect insight in projected future changes of AECPG delivery, we meta-synthesized existing scenarios that include provision of AECPGs. Meta-synthesis integrates qualitative studies in a related area, summarizing key elements across studies (Walsh and Downe 2005). To ensure relevance to national-level stakeholder visions, we only included scenarios focused on the Dutch agricultural sector in its entirety, written by academia or governmental knowledge institutes after 2017, describing a long-term (2050 or beyond) perspective.

To collect academic studies, we searched the Scopus database with the following query: TITLE-ABS-KEY (scenario* AND Dutch AND agriculture) AND PUBYEAR > 2017 AND SUBJAREA (agri) OR SUBJAREA (envi). This gave 12 results, of which two met the full criteria. To collect studies from governmental knowledge institutes, we searched Google Scholar, with the search query (scenario* landbouw 2050 OR scenario* landbouw Nederland OR scenario* landbouw 2050 Nederland AND beyond 2017). This gave 1260 hits, of which the first 100 were scanned and 3 met the inclusion criteria. Snowball sampling using the initial results until similar themes and results reappeared in different scenarios and no novel themes emerged (saturation) yielded an additional 4 studies, resulting in 6 studies in total that describe 9 scenarios.

Analyzing the existing scenarios revealed four relevant themes that defined the meta-scenarios: productivity/sustainable intensification, meadow bird conservation, region-based approaches, and nature inclusive agriculture. The scenarios were meta-synthesized by integrating storylines in terms of proposed measures, key characteristics and mentioned or implied AECPGs (Supplementary material).

We coded AECPG supply in all input scenarios. When indifferent (coded zero), the AECPG was not mentioned in the scenario, or mentioned in the problem statement but not as a specific aim. Active conservation (0.5) was assumed if the scenario explicitly aimed at preserving current levels of AECPG delivery, or if the scenario adhered to existing regulations around an AECPG. Improvement (1) was assigned if enhancing AECPG delivery was explicitly mentioned or clearly implied, or if the scenario quantitatively predicted improvement of AECPG delivery. The final score per AECPG for each of the four meta-scenarios was obtained by calculating the average scores and rounding to 0, 0.5 or 1.

### Comparing expected and desired AECPG delivery

AECPG supply and expressed desire were compared using the agreement calculation from Helfenstein et al. (2022). For each scenario, expected change and desired delivery were compared by calculating the absolute difference (Eq. 1):

1 $$Difference\; between\; expected\; and\; desired\; change \left( {DEA} \right) = \mathop \sum \nolimits_{i = 0}^{n} abs\left( {e_{i } - d_{ij} } \right)*w_{ij}$$

where *e*_*i*_ is the expected change of AECPG i, *d*_*ij*_ is the desired delivery of AECPG i by stakeholder j, and *w*_*ij*_ is the weight of the AECPG i for stakeholder j. For each AECPG, potential agreement between expected change and desired delivery was calculated using Eq. (2):

2 $$Potential\; agreement \left( {PA} \right) = \mathop \sum \nolimits_{i = 0}^{n} w_{ij}$$

An agreement score between expected change in a scenario and desired delivery by a stakeholder group was calculated following (Eq. 3).

3 $$Agreement = 100 * \frac{{\left( {PA - DEA} \right)}}{PA}$$

The agreement scores were summarized per stakeholder group by calculating the mean agreement of all stakeholders in a group, rounded to a one-decimal percentage. Agreement was classified into five classes: high agreement (> 80%), agreement (60–80%), moderate (40–60%), disagreement (20–40%) and strong disagreement (< 20%). The scenario with the highest agreement was considered optimal.

Two sensitivity analyses were performed. As the scoring of desired change and expected delivery is subjective and can influence agreement levels, we analyzed the impacts of both scorings on the outcomes. First, we quantified the influence of the weights assigned to AECPGs for different stakeholders, by changing all weights with value 2 to 1 before calculating agreement. This analysis was done to explore the effect of the classification of the weights in three classes, following Helfenstein et al. (2022). Second, we explored the impact of the AECPG delivery in the explorative scenarios into three classes by changing all scores 0.5 to 0 before calculating agreement. This sensitivity analysis was performed to explore the effect of scoring the scenarios.

## Results and discussion

### Stakeholder desire for AECPG delivery

**Fig. 2:**
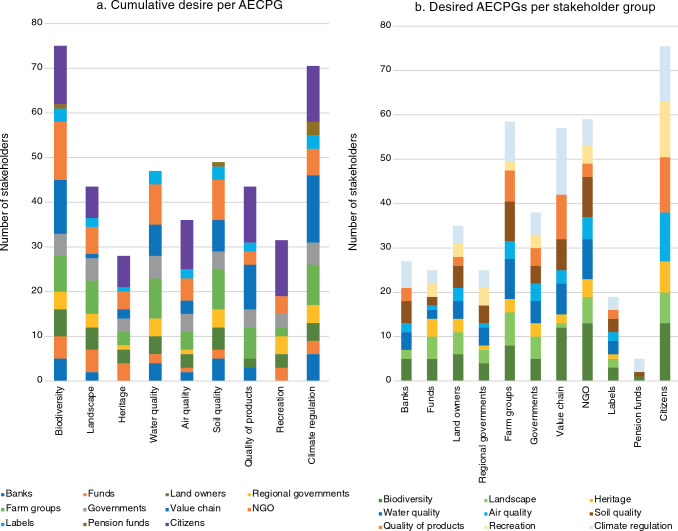
AECPG desire **a** cumulative per AECPG and **b** per stakeholder group. The length of the bar shows the number of stakeholders that expressed desire for the AECPG. In total 83 stakeholders were included

Every stakeholder group expressed desire for at least one AECPG (Fig. 2), and priorities for AECPGs provision clearly varied between stakeholder groups (Table 2; Supplementary material).

**Table 2:** Mean (Standard error) AECPG desire of all stakeholder groups for all AECPGs. Desire ranges from zero (no desire expressed) to one (high desire expressed)

Stakeholder group	*N*	Public goods								
		Biodiversity	Aesthetic landscape quality	Heritage	Water quality	Air quality	Soil quality	Quality of products	Recreation	Climate regulation
Banks	6	0.83 (0.17)	0.33 (0.21)	0 (0)	0.67 (0.21)	0.33 (0.21)	0.83 (0.17)	0.5 (0.22)	0 (0)	1 (0)
Funds	5	1 (0)	1 (0)	0.8 (0.2)	0.4 (0.24)	0.2 (0.2)	0.4 (0.24)	0 (0)	0.6 (0.24)	0.6 (0.24)
Landowners	6	1 (0)	0.83 (0.17)	0.5 (0.22)	0.67 (0.21)	0.5 (0.22)	0.83 (0.17)	0.33 (0.21)	0.5 (0.22)	0.67 (0.21)
Value chain	18	0.67 (0.11)	0.06 (0.06)	0.11 (0.08)	0.39 (0.12)	0.17 (0.09)	0.39 (0.12)	0.56 (0.12)	0 (0)	0.83 (0.08)
Governments	7	1 (0)	0.86 (0.14)	0.57 (0.2)	1 (0)	0.71 (0.18)	0.86 (0.14)	0.57 (0.2)	0.71 (0.18)	1 (0)
NGOs	13	1 (0)	0.46 (0.14)	0.31 (0.13)	0.69 (0.13)	0.38 (0.14)	0.69 (0.13)	0.23 (0.12)	0.31 (0.13)	0.46 (0.14)
Labels	3	1 (0)	0.67 (0.33)	0.33 (0.33)	1 (0)	0.67 (0.33)	1 (0)	0.67 (0.33)	0 (0)	1 (0)
Pension funds	3	0.33 (0.33)	0 (0)	0 (0)	0 (0)	0 (0)	0.33 (0.33)	0 (0)	0 (0)	1 (0)
Farmer organizations	9	0.8 (0.11)	0.75 (0.11)	0.3 (0.16)	0.9 (0)	0.4 (0.17)	0.9 (0)	0.7 (0.14)	0.2 (0.14)	0.9 (0)
Citizens	13	1 (0)	0.54 (0.07)	0.54 (0.07)	0 (0)	0.85 (0.07)	0 (0)	0.96 (0.04)	0.96 (0.04)	0.96 (0.04)

At least one stakeholder in each group expressed a desire for ‘biodiversity’ or ‘climate regulation’, and these were also the AECPGs most frequently mentioned in stakeholder policies and visions. This might reflect the urgency of climate change and biodiversity in national and international policy agendas, e.g., through the Paris Agreement, Convention on Biological Diversity, national climate agreement and “Deltaplan biodiversiteitsherstel” (van Bodengraven 2019). We found a higher desire for biodiversity than for climate regulation, contrary to Legagneux et al. (2018), but in line with a recent study of agri-environmental contracts for the delivery of public goods in the Netherlands (Harmanny et al. 2024). Climate regulation might be perceived as a global problem related to industry and pollution, whereas biodiversity has a more local relation to agriculture (De Boer and Aiking 2021). Also, although younger generations acknowledge the role of human activities in climate change, they poorly understand its relation to agriculture and the food system (Bogueva and Marinova 2022).

Across all stakeholder groups, ‘heritage’ and ‘recreation’ showed the lowest expressed desire. This is surprising given the explicit attention for landscape heritage in agricultural policy (Simoncini et al. 2019), and given the importance of heritage for explaining revealed preference among recreationists (Tieskens et al. 2018). For stakeholders other than end users (citizens), heritage is intangible (Garcia-Martin et al. 2017), and stakeholders might be physically or socially distanced from natural heritage (Jaligot et al. 2019). For recreation, utility to the stakeholder might play a role, as also suggested by citizen’s desire for recreation and heritage that is in the same order of magnitude as for other AECPGs (Table 3). Aesthetic landscape quality was predominantly desired by stakeholder groups owning, working or conserving land, such as farm groups and NGOs. Quality of products was desired mostly by stakeholders consuming (citizens), producing (farmer’s organizations) and selling (supermarkets) products. Biodiversity, soil and water quality were most relevant to farmers because of their utility to production (Zoderer et al. 2019; LTO Vrouw en Bedrijf, personal communication, July 2022). Overall, these results support the utility hypothesis, showing the influence of stakeholder connection to a certain AECPG on its demand (Garcia-Martin et al. 2017; Zoderer et al. 2019).

**Table 3:** Citizen’s AECPG desire by sub groups (% of respondents indicating desire for AECPG). Full data can be found in the Supplementary material

	Air Quality (%)	Water Quality (%)	Soil Quality (%)	Aesthetic landscape quality (%)	Climate regulation (%)	Quality of products (%)	Biodiversity (%)	Recreation (%)	Heritage (%)
Total	41	19	12	30	62	44	54	41	42
Gender									
Men	39	20	11	29	63	42	53	40	40
Women	44	18	13	30	61	45	54	42	43
Political orientation									
Left	45	18	13	32	71	40	59	46	39
Centre	28	21	11	30	57	43	51	39	45
Right	29	17	12	27	53	50	47	36	41
Age group									
Young (15–24)	46	16	6	25	71	36	47	31	38
Young adult (25–39)	43	22	11	38	59	47	55	38	32
Senior adult (40–54)	28	20	13	35	61	45	55	44	42
Senior (55+)	41	19	14	23	62	43	54	45	49
Living environment									
Rural	39	18	11	21	59	45	53	42	42
Smal/mid size town	40	20	13	32	62	42	53	42	40
Large town	48	21	12	41	66	43	56	39	44

No widespread desire for water and soil quality was observed among citizen groups (Table 3). This might be an artifact of the Eurobarometer data, or related to low tangibility (Vrščaj et al. 2008).

In line with Rogge et al. (2007), fewer rural than urban citizens expressed desire for aesthetic landscape quality (Table 3). Howley et al. (2012) however observed that rural dwellers were more likely to prefer traditional farm landscapes than urban dwellers. The desire for heritage increased with age (Table 3). Generations growing up in an urbanized country might have different perceptions and expectations of natural heritage than older generations, due to a ‘shifting baseline syndrome’ (Jones et al. 2020).

Within the stakeholder groups ‘citizens’, ‘farmer organizations’ and to a lesser extent ‘pension funds/insurers’, AECPG desires were highly homogenous while heterogeneity was higher for labels, funds, and value chains (Table 2). All included funds and insurers follow the same neoliberal ideology. They were, however, also the groups with the lowest sample size (3). The homogeneity among citizens’ AECPG desire was surprising and contrasting other studies (Castro et al. 2011). While AECPG desires for all other stakeholder groups were extracted from individual stakeholder visions, citizens’ AECPG desire was quantified based on survey data that inventoried visions of all different citizen sub groups in a consistent way. This different sampling procedure might have led to a relative underestimation of the heterogeneity of citizens’ visions. The value-chain group was also the most heterogeneous group in terms of stakeholders, ranging from supermarkets to fertilizer companies. While labels all operate from similar nature-focused ideologies, they range from incremental (planet proof) to transformative foci, potentially explaining the high heterogeneity. Homogenous stakeholder categorization can minimize assumptions about stakeholder interests and enhance inclusivity (Arnette et al. 2010). Therefore, future research should further investigate the currently heterogenous groups, to identify more homogenous clusters (Table 4).

**Table 4:** Summary of the meta-scenarios. The Supplementary material provides a full description

Topic	Meta-scenario			
	Productivity	Integral	Meadow bird	Regional
Agricultural production	Intensification and scale enlargement under climate regulation	Extensification. Lower production in circular agriculture	Extensification	Intensification and extensification
Zonation	Land sparing	Extensification throughout the Netherlands	Extensification in meadow bird breeding areas throughout the Netherlands	Extensification around N2K and in sand region, intensification in clay region
AECPG supply by agriculture	Low	High	Medium	High
Nature protection	Outside agricultural zones	Holistic nature protection throughout agricultural landscape	Strong focus on conservation of meadow bird populations and habitats	Holistic nature protection in extensive zones
Farm income diversification	Emission targets, carbon trading and CCS	Added product value, payment for AECPGs, sustainable energy, health care on farm	Subsidies for landscape management	Added product value, payment for AECPGs, sustainable energy, emission targets, carbon trading and CCS

In the polarized Dutch agricultural debate, farmers are sometimes depicted as ignorant or negligent towards sustainability (van Vuuren-Verkerk et al. 2021). However, the farm groups had a clear demand for all AECPGs. This was confirmed in the interviews: farmers feel responsible for maintaining land quality and delivering AECPGs (LTO Vrouw en Bedrijf, personal communication). Nonetheless, AECPG delivery requires agricultural business models with fair remuneration (Barghusen et al. 2021), which depends on mature payment schemes for individual AECPGs.

### Expected AECPG supply in meta-scenarios

The themes of the four identified meta-scenarios connect to broader research and policies. Intensification has been the main development in Dutch agriculture over the last decades, and continuation of highly productive agricultural systems is widely considered as a business-as-usual (Skevas et al. 2018). Meadow bird conservation is a key theme in policy, with different governance arrangements facilitating and financing nature conservation by farmers (Runhaar and Polman 2018), among others because of the high cultural value of meadow birds (e.g., black-tailed godwit). In scenarios with an ‘integral’ theme, a drastic national transition to nature inclusive agriculture is described. Finally, the ‘regional’ approach combines extensive and intensive agriculture differentiated by area. This aligns with new CAP legislation, where multiple responsibilities will shift to regional level (Netherlands Enterprise Agency 2021).

Dutch scenarios are embedded into the broader European and global context. For example, global survival of the black-tailed godwit requires habitat conservation in the Netherlands, its key breeding area (Gill et al. 2007). This global responsibility of the Netherlands to sustain the species emphasizes the relevance of the meadow bird scenario. The regional scenario aligns with EU-level scenarios focusing on a higher level of regionalization (e.g. Mouchet et al. 2017). If intensive agriculture disappears from the Netherlands under the integral scenario, this might cause displacement and land grabbing elsewhere (Hossein et al. 2016). Although the productivity scenario would result in limited AECPG supply, significant reductions in global agricultural emissions are feasible when precision agriculture is applied in line with the 2-degree target. Such a scenario relying on skill-biased technology could, however, cause wage inequality within the country and shift power towards capital-rich stakeholders (O’Neill et al. 2017). Moreover, a productivity scenario would only support biodiversity if more land comes under conservation management, i.e., if higher productivity leads to land sparing (Springmann et al. 2018). Focusing solely on agricultural transformations demonstrates that a land sharing approach leads to broader AECPG supply instead (Table 5).

**Table 5:** Expected change matrix of the AECPGs for each meta-scenario. The explorative scenario studies used as input for the meta-scenario are added in brackets. 0: no action mentioned or implied enhancing supply of the AECPG. 0.5: active conservation or minimal increase of AECPG supply. 1: demonstrable enhancement of AECPG supply

Meta-scenario (sources)	Biodiversity	Aesthetic landscape quality	Heritage	Water quality	Air quality	Soil quality	Quality of products	Recreation	Climate regulation
Regional (College van Rijksadviseurs 2020; Bakker 2021)	1	1	0.5	1	0.5	1	0.5	1	1
Productivity (Lesschen et al. 2020; Gonzalez-Martinez et al. 2021)	0	0	0	0.5	0.5	0	0	0	1
Meadow bird (Melman and Sierdsema 2017; van Hinsberg et al. 2020a, b)	1	1	1	0.5	0	0.5	0	0.5	0.5
Integral (Lesschen et al. 2020; Gonzalez-Martinez et al. 2021; Breman et al. 2022)	1	1	0.5	1	0	1	0.5	0.5	1

### Agreement between AECPG supply and desire

**Fig. 3:**
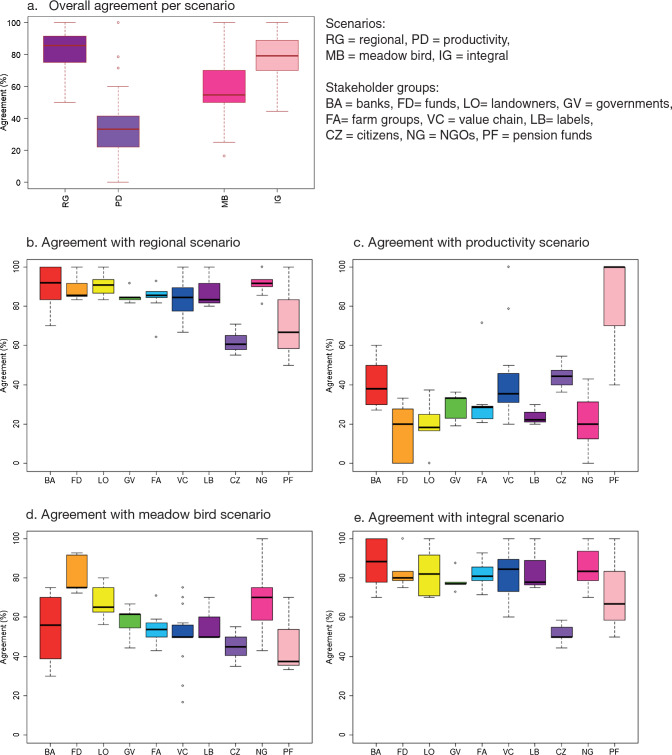
**a** Agreement of all stakeholder visions with the scenarios in %. **b**–**e** Agreement between observed and desired change broken down for each stakeholder group. Each point shows a stakeholder

The regional scenario showed the highest (83%) agreement between AECPG supply and desire (Fig. 3a; Table 6), with the highest agreement for all stakeholder groups except pension funds (Fig. 3b). The integral scenario had the second highest agreement (78%), which is not surprising given the similar AECPG supply (Table 5). The focus on popular themes, like biodiversity and climate regulation, might be a cause for this high agreement. While the meadow bird scenario did not stand out as the best scenario for any stakeholder group with a moderate agreement score of 58%, the scenario aligns well with the demands of “funds”. As funds are financially powerful stakeholders that address individual farmers, this might be a credible sub-scenario on small scales. The productivity scenario showed the lowest (34%) agreement between supply and desire. Especially stakeholder groups with environmental ideology scored low, e.g. NGOs, but the scenario also shows a strong disagreement with “funds”. This might reflect widespread doubts about the sustainability of the Dutch agricultural sector. Agreement with this scenario is primarily due to the high climate regulation supply targeted in this scenario. However, climate regulation measures in this scenario might focus on technological solutions, which are only desirable to a limited group of stakeholders. This was not considered in detail in the meta-scenarios, because of lack of detailed information.

**Table 6:** Overview of sensitivity analysis. Full results are in the Supplementary material

	Scenario			
	Regional	Productivity	Meadow bird	Integrated
Baseline agreement				
Overall agreement	83	34	57	78
Max. agreement (stakeholder group)	92 (NGOs)	80 (Pension funds)	81 (Funds)	86 (NGOs)
Min. agreement (stakeholder group)	61 (Citizens)	16 (Funds)	45 (Citizens)	51 (Citizens)
Sensitivity analysis: simplified weights				
Overall agreement	83	34	57	77
Max. agreement (stakeholder group)	90 (NGOs)	78 (Pension funds)	78 (Funds)	85 (Banks)
Min. agreement (stakeholder group)	61 (Pension funds)	16 (Funds)	42 (Pension funds)	51 (Citizens)
Sensitivity analysis: simplified delivery				
Overall agreement	73	27	38	68
Max. agreement (stakeholder group)	85 (NGOs)	80 (Pension funds)	65 (Funds)	82 (Banks)
Min. agreement (stakeholder group)	43 (Citizens)	11 (NGOs)	22 (Pension funds)	34 (Citizens)

The outliers in the alignment with the productivity scenario (Fig. 3c) were pension funds and fertilizer producers. The scenario aligns with the interest of fertilizer producers as it meets legal (global) emission targets, but maintains a market for their product. This causes a risk of greenwashing, as it benefits their corporate social and climate responsibility reporting at the cost of broader nature inclusivity (Mahoney et al. 2013). For the regional scenario, a large pension fund, a dairy farm union, and citizens were the outliers (Fig. 3). This scenario would require a drastic system change, which contrasts common agri-food system configurations in which government subsidies and corporate policies create a lock-in (Williams et al. 2023). The dairy farm union experienced decentralized nature policy so far as unclear and unbeneficial and thus rejects further decentralization. For citizens, the low agreement with the regional and integral scenarios might be due to the emphasis on soil and water quality in the scenario, while this is not a key AECPG to citizens (Table 2).

In the sensitivity analyses, simplifying the AECPG supply in the scenarios lowered agreement levels for all stakeholders for the ‘meadow bird’ scenario (Table 6; Supplementary material). This confirmed the strong influence of climate regulation, as there would be no supply of climate regulation under this simplification. Although agreement percentages changed, the order of magnitude was stable. Agreement levels were rather robust towards the weight assigned to AECPGs by stakeholders (Table 6), suggesting that weight assignment might not enrich the results.

While the results suggest that all stakeholder groups would agree on transitioning towards the regional scenario, there are limitations. First, stakeholder data consists largely of self-reported communications. With increasing corporate social responsibility reporting, sustainability can become a token in showing corporate responsibility, with the danger of greenwashing (Mahoney et al. 2013). Second, a conflict of interest might exist between the inherent character of a stakeholder (for example its core-business) and the delivery of AECPGs. For example, soil health is vital for biodiversity and sustainable agriculture. However, Dutch agriculture strongly depends upon (chemical) fertilizers, which have adverse effects on physicochemical and biological properties of soil and water, and contribute to nitrogen emissions (RIVM 2020). Also, the proposed CAP National Strategic Plan and farmer’s responses to it shows that discourse and practice do not always align (LTO Vrouw en Bedrijf, personal communication, July 2022). Long-term commitments and raising awareness about AECPGs in all layers of society might improve this alignment.

### Discussion of the methodology

While our exploration and quantification of agreement between desired and expected change across society in AECPG delivery included a group of stakeholders as complete as possible, our study also has limitations. First, the Dutch agricultural system is not closed, meaning that a stakeholder inventory could never be exhaustive. Many food system actors are transnational, operating on multiple levels (Clapp and Fuchs 2009). Overlooking stakeholders means overlooking realities (Creswell 2013), but, complete reality could never be captured, as stakeholders are ever evolving (Verkerk et al. 2018). Therefore, there is a continuous need for research involving stakeholders, including non-humans and media actors which were not covered, but influential (Rust et al. 2021).

While we assumed independency between the scenarios and stakeholder visions, there might be inference. Stakeholders respond to perceptions of their environment, meaning that information about future scenarios will influence their vision, however reliant on access to information (el Bilali and Allahyari 2018). Bias was minimized by using the most recently published scenarios, and ensuring that scenarios and stakeholder visions were as independent as possible, by not considering the authors (researchers) as stakeholders. Additionally, triangulation during the interviews showed that the agroecological and women farm organizations recognized the meta scenarios and considered them plausible (LTO Vrouw en Bedrijf; Toekomstboeren, personal communication, July 2022).

When scoring AECPG desires, we used a three-level classification for citizens and distinguished two levels for other stakeholders. This might have inflated the AECPG desires of citizens. Next, we analyzed AECPGs as individual categorical variables. In reality, AECPGs might interact (Zoderer et al. 2019). Most importantly, synergies exist between and amongst multiple regulating and cultural services (Zoderer et al. 2019). A modelling study quantifying these relations could build on this study and extend the concept of synergies to AECPGs.

Finally, a central theme in Dutch agricultural debate that was not addressed is nitrogen reduction, as reduction targets are still being developed and not available (Staghouwer 2022).

### Pathways towards optimal AECPG provision

Comparing the identified meta-scenarios with European scale scenarios (Mitter et al. 2020) suggests that scenarios with enhanced AECPG provision (regional scenario and integral scenario) might emerge in a sustainability scenario, with tightened pro-environmental policies, abolishment of income support, and better-connected markets (Eur-Agri-SSP2). This scenario also assumes reduced climate mitigation and adaptation challenges, reducing the desire for the climate regulation AECPGs. Production-oriented scenarios with increased pressure on AECPG delivery, similar to our “production” scenario (Table 5) are common in European-scale scenario studies, e.g., this aligns with Mitter et al.’s (2020) Eur-Agri-SSP3 and with the production-oriented scenario from Mouchet et al. (2017).

Altogether, the regional scenario aligns best with the AECPG demands of a broad range of stakeholders. This scenario sketches a drastic transformation of the Dutch rural area. Moving towards the regional scenario will take decades, with considerable spatial implications (College van Rijksadviseurs 2020; Bakker 2021). The scenario might require farm termination or relocation (Bakker 2021), and remaining farmers will have to diversify their income.

A long-term policy with clear targets on AECPG provision is a prerequisite (College van Rijksadviseurs 2020; LTO Vrouw en Bedrijf, personal communication, July 2022). This can provide the clarity and foster the alignment of goals that is needed to foster innovation (Williams et al. 2023), and the ability of farmers to implement nature conservation measures might benefit from ambitious greening requirements (Runhaar et al. 2016). Therefore, a clear national policy would set the ground for transition. However, the Dutch *Polder* model not necessarily represents all voices equally. Corporate value-chain actors have instrumental, structural and discursive power, with which they influence farmers, governments and citizens (Clapp and Fuchs 2009). When such actors are structurally engaged in the policy arena, they tend to supplement or replace state actors (Clapp and Fuchs 2009), with the risk of multiplying their power. For example, a fertilizer producer will have instrumental and structural power, but one might question whether they are entitled discursive power in a scenario where limits to fertilizer use support AECPG provision to all stakeholders, at the cost of their preferences and benefits.

Secondly, farm diversification requires a financial basis for remaining farms, and might require a change towards a more agroecology-oriented farming system across the Netherlands. While this is supported by the new CAP (Netherlands Enterprise Agency 2021), business models or contracts that guarantee and finance long-term AECPG provision are needed. Labelling stakeholders can add value to products, indicating heritage, product quality and landscape aesthetics supply (García-Martín et al. 2021). Upon relocation and diversification as well as for an agroecology transition, land availability is a crucial factor, causing challenges but also opportunities for different stakeholders. Addressing land availability whilst involving investors like pension funds, lotteries and banks could be supported through a “community land trust” (Bakker 2021). Alternatively, a public land management institution could support land availability (College van Rijksadviseurs 2020). This change might be supported by fostering structural power of marginal stakeholders that advocate such a transition.

Third, a broader change in the Dutch food system is required, including changes in the attitude of citizens, and change of communication across value chains. Current citizens’ visions might be unrealistic; desiring extensively produced food, that is cheap and sustainable, as well as large homes with gardens, is not compatible with land availability in the Netherlands. While citizens primarily see a role for farmers and food manufacturers in food systems transformations (European Commission 2020), they also are responsible for a transition towards more sustainable consumption themselves. Aligning their consumption behavior with their citizen values regarding sustainability (Lehner 2013) might lever a broader transition. “A little chauvinism” (LTO Vrouw en Bedrijf) by not only relying on regional landscapes for public good supply but also for food production, could provide financial support for this transition. Also, retail has power in shaping consumer opinion (Schulp et al. 2022). Proper communication of these developments to society could influence citizen opinion and create opportunity for local understanding and initiatives.

Although this scenario seems ambitious, it would not be the first quick and drastic change in landscape policy. The forestry sector transformed within a decade from a timber supplier to a multifunctional nature area, providing leisure opportunities and natural heritage (Veenman et al. 2009). A discursive shift, leading to a more public debate, was the main driver (Veenman et al. 2009). The heated agricultural debate might therefore foreshadow a food system transformation. If discourse, power, rules and actors align, and stakeholders practice what they preach, the regional scenario could be achieved.

## Conclusion

Based on an inventory of normative visions of 83 stakeholders from ten groups, this study provides a comprehensive, national-scale, nuanced overview of societal desires for agri-environmental-climate public goods. Biodiversity and climate regulation were desired most frequently, and by all stakeholder groups, and AECPG utility to a stakeholder influences a stakeholder’s desire for the AECPG. Societal desire for AECPGs is best fulfilled under a scenario of large scale extensification, where intensive production is limited to designated zones. This demonstrates that sustainable AECPG provision requires a transition towards large-scale nature-inclusivity, that has considerable spatial and policy implications. Choosing the scenario with the highest agreement is a first but important step for policy makers to get everyone on board in a transition to sustainable agriculture.

### Supplementary Information

Below is the link to the electronic supplementary material.

Supplementary file1 (XLSX 157 KB)

## Data Availability

The datasets based on open data generated during the current study are added to the paper as supplementary material. Other data are available from the corresponding author on reasonable request.

## Footnotes

1 www.nieuweoogst.nl.
